# The anterior thalamic nuclei and nucleus reuniens: So similar but so different

**DOI:** 10.1016/j.neubiorev.2020.10.006

**Published:** 2020-12

**Authors:** Mathias L. Mathiasen, Shane M. O’Mara, John P. Aggleton

**Affiliations:** a School of Psychology, Cardiff University, 70 Park Place, Cardiff, CF10 3AT, Wales, UK; b School of Psychology and Institute of Neuroscience, Trinity College, Dublin, Ireland

**Keywords:** Cingulate cortex, Hippocampus, Mammillary bodies, Memory, Prefrontal cortex, Retrosplenial cortex, Spatial memory, Subiculum, Thalamus

## Abstract

Two thalamic sites are of especial significance for understanding hippocampal – diencephalic interactions: the anterior thalamic nuclei and nucleus reuniens. Both nuclei have dense, direct interconnections with the hippocampal formation, and both are directly connected with many of the same cortical and subcortical areas. These two thalamic sites also contain neurons responsive to spatial stimuli while lesions within these two same areas can disrupt spatial learning tasks that are hippocampal dependent. Despite these many similarities, closer analysis reveals important differences in the details of their connectivity and the behavioural impact of lesions in these two thalamic sites. These nuclei play qualitatively different roles that largely reflect the contrasting relative importance of their medial frontal cortex interactions (nucleus reuniens) compared with their retrosplenial, cingulate, and mammillary body interactions (anterior thalamic nuclei). While the anterior thalamic nuclei are critical for multiple aspects of hippocampal spatial encoding and performance, nucleus reuniens contributes, as required, to aid cognitive control and help select correct from competing memories.

## Highlights

• Despite overlapping cortical connectivity, different dorsal/ventral gradients.
• Common domains of function but different contributions.
• Primacy of anterior thalamic - retrosplenial interactions.
• Primacy of nucleus reuniens - frontal interactions.
• Contrasting patterns of hippocampal - thalamic interactivity.

## 1 Introduction

**Fig. 1:**
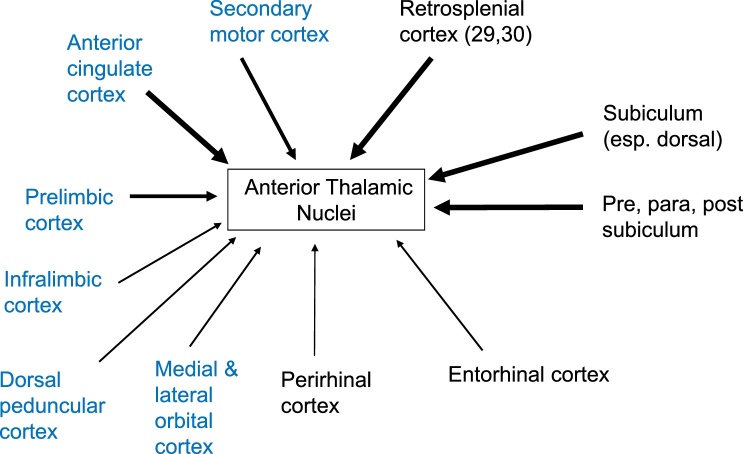
Cortical (and allocortical) inputs to the anterior thalamic nuclei (ATN). The sites in blue have few, if any, direct projections to the hippocampal formation. The thickness of the line indicates the scale of the input.

**Fig. 2:**
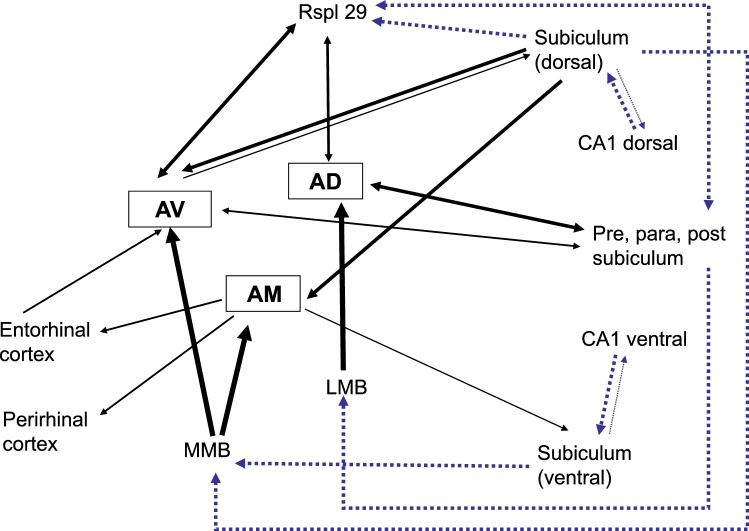
Schematic diagram of the main connections of the hippocampal formation and parahippocampal region with the anterior thalamic nuclei (ATN). The thickness of the line indicates the scale of the input, while the lightest connections are not shown. The indirect connections (dotted blue lines) involving retrosplenial cortex and the mammillary bodies are shown, reflecting their significance for anterior thalamic function. Note, not all connections are shown, just those most pertaining to the ATN. Abbreviations: AD, anterodorsal thalamic nucleus; AM, anteromedial thalamic nucleus; AV, anteroventral thalamic nucleus; c, cortex; LMB, lateral mammillary nucleus; MMB, medial mammillary nucleus; para, parasubiculum; post, postsubiculum; pre, presubiculum; Rspl, retrosplenial cortex, area 29.

Within the thalamus, the anterior thalamic nuclei and nucleus reuniens stand out because of their dense, direct interactions with the hippocampus and frontal cortices. In fact, the similarities in their connections extend much further. Remarkably, almost every cortical or subcortical site that projects to the rat anterior thalamic nuclei also appears to project to nucleus reuniens (Fig. 1, Fig. 2). Furthermore, electrophysiological recordings show that both thalamic areas contain spatially-responsive neurons. Reflecting these shared properties, lesions in both sites disrupt spatial tasks known to depend on the integrity of the rodent hippocampal formation. Despite these similarities, this review highlights how these two thalamic sites are, in fact, quite dissimilar. Their differences, which begin with the details of their respective connections, lead to distinct predictions about their respective functional contributions, even though they are engaged in overlapping domains.

The spatial and episodic memory functions of the anterior thalamic nuclei are largely set by their hippocampal and retrosplenial interconnections, as well as their dense mammillary body inputs. Consequently, the rodent anterior thalamic nuclei are vital for an array of ongoing spatial and contextual learning processes, reflecting their role in encoding and retrieving multiple aspects of space and navigation. For this reason, they maintain features of ‘first’ order thalamic nuclei, which receive ascending peripheral information from subcortical sources (Sherman and Guillery, 1996; Sherman, 2007), e.g., from the mammillary bodies.

In contrast, the functions of nucleus reuniens are largely governed by its numerous prefrontal cortex connections, alongside its dense projections to the hippocampus. Nucleus reuniens also has a much broader array of subcortical connections than the anterior thalamic nuclei. The contributions of nucleus reuniens become most apparent when memory demands are increased (e.g., high interference or long retention delays) or when greater cognitive flexibility is required (Cholvin et al., 2013; Griffin, 2015; Barker and Warburton, 2018). Consequently, the effects of nucleus reuniens lesions can be less immediately evident than those of anterior thalamic lesions.

The behavioural analysis largely concentrates on rat spatial learning, given the numbers of relevant studies. In view of this focus we should, at the outset, acknowledge one well-established difference between the anterior thalamic nuclei and nucleus reuniens. This difference concerns their relative importance within the head-direction system (Taube, 1995, 2007). Not only do both the anterodorsal and anteroventral thalamic nuclei contain many head-direction cells (Taube, 1995; Tsanov et al., 2011) but lesions involving these nuclei stop head-direction information from reaching the parahippocampal region (Calton et al., 2003). Nucleus reuniens, while also containing some head-direction cells (Jankowski et al., 2014) does not appear to be a critical relay for downstream sites. It might, therefore, be supposed that their relative importance within the head-direction system can explain differences in the outcome of nucleus reuniens and anterior thalamic lesions on spatial tests. In fact, the anteroventral and anteromedial thalamic nuclei make additional contributions to spatial learning that are seemingly distinct from conveying head-direction information (Aggleton et al., 1996, 2010; Byatt and Dalrymple-Alford, 1996; Van Groen et al., 2002; Aggleton and Nelson, 2015). For this reason, the loss of head-direction information appears insufficient to account for the severity of the spatial deficits following anterior thalamic lesions (Vann and Dillingham, 2019).

Before discussing relevant evidence in more detail is it helpful to establish various terms. The anterior thalamic nuclei are principally composed of the anteromedial, anteroventral, and anterodorsal nuclei. Although these three nuclei have many similar connections, numerous topographic differences ensure that the fine details vary. These same topographies highlight the potential for specialisation within each of the three thalamic nuclei. While the laterodorsal thalamic nucleus shares many properties with the anterior thalamic nuclei, it lacks mammillary body inputs. For this reason, it is treated as distinct. In the rodent brain, a separate interanteromedial nucleus is recognised at the midline.

Nucleus reuniens, which is also located on the midline, lies below the interanteromedial nucleus and the rhomboid nucleus. Nucleus reuniens is not uniform as its various connections show different gradients within the structure (Herkenham, 1978; Van der Werf et al., 2002; McKenna and Vertes, 2004). While the terms hippocampal formation and hippocampal refer to the dentate gyrus, CA fields, and subiculum (Burwell and Witter, 2002), the presubiculum, postsubiculum, parasubiculum, and entorhinal cortex all comprise parts of the parahippocampal region. It should also be remembered that the rodent ventral (or ‘temporal’) hippocampus is homologous with the primate anterior hippocampus, while the dorsal (or ‘septal’) hippocampus is homologous with the primate posterior hippocampus.

## 2 Connectivity

### 2.1 Anterior thalamic nuclei

All three anterior thalamic nuclei have dense reciprocal connections with the retrosplenial cortex while the anteromedial and anteroventral nuclei have additional reciprocal connections with the anterior cingulate cortex (Seki and Zyo, 1984; Van Groen and Wyss, 1990a, 1992, 2003; Shibata, 1998; Van Groen et al., 1999; Shibata and Naito, 2005; Wright et al., 2013; see Fig. 1). Other areas with reciprocal anteromedial and anteroventral nuclei connections include the secondary motor cortex and entorhinal cortex, while lighter connections are associated with the medial orbital and infralimbic cortices (Shibata, 1996; Shibata and Naito, 2005; Wright et al., 2013). Despite the dense inputs from the prelimbic cortex to the anteromedial and anteroventral nuclei, it is the interanteromedial nucleus that gives rise to the densest projections to the prelimbic cortex, with the anteromedial nucleus also contributing (Varela, et al., 2014; Van Groen et al., 1999).

The hippocampal formation is also reciprocally connected with the anterior thalamic nuclei (Meibach and Siegel, 1977a,1997b; Swanson, 1978; Shibata, 1993; Van Groen and Wyss, 1995; Van Groen et al., 1999; see Fig. 2). Dense hippocampal projections arise from deep cells within the dorsal and intermediate subiculum to reach the anteromedial and anteroventral nuclei (Meibach and Siegel, 1977b; Christiansen et al., 2016), nuclei that both project back upon the subiculum (Shibata, 1993; Van Groen and Wyss, 1995; Van Groen et al., 1999). Meanwhile the anterodorsal thalamic nucleus has reciprocal connections with the postsubiculum and presubiculum (Van Groen and Wyss, 1990b,c; Van Groen and Wyss, 1995; see Fig. 2).

Among its subcortical afferents, those from the mammillary bodies are pre-eminent (Seki and Zyo, 1984; Shibata, 1992). Almost every mammillary body neuron is thought to innervate the anterior thalamic nuclei (Takeuchi et al., 1985), with the various anterior thalamic nuclei receiving inputs from different mammillary subregions (Seki and Zyo, 1984; Shibata, 1992). Some mammillary body projections to the anterior thalamic nuclei bifurcate to also innervate Gudden’s tegmental nuclei (Takeuchi et al., 1985; Hayakawa and Zyo, 1989). Other appreciable subcortical inputs to the anterior thalamic nuclei arise from the thalamic reticular nucleus and the laterodorsal tegmental nucleus, while lighter inputs originate from the pedunculopontine tegmental nucleus and the median raphe nucleus (Cornwall et al., 1990a; Vertes, 1991; Gonzalo-Ruiz et al., 1995a,b; Gonzalo-Ruiz and Lieberman, 1995; Lozsadi, 1995; Vertes et al., 1999).

### 2.2 Nucleus reuniens

Like the anterior thalamic nuclei, nucleus reuniens has reciprocal connections with frontal cortices, the anterior cingulate cortex, retrosplenial cortex, the subiculum, perirhinal cortex, and entorhinal cortex (Herkenham, 1978; McKenna and Vertes, 2004; Vertes et al., 2006; Cassel et al., 2013; Varela et al., 2014; Mathiasen et al., 2019, Fig. 3). Its afferent connections from the prelimbic, infralimbic, rostral anterior cingulate cortex, dorsal peduncular, lateral and medial orbital cortex are particularly dense, while those from retrosplenial cortex are much lighter (McKenna and Vertes, 2004; Mathiasen et al., 2019). Meanwhile, many of its hippocampal inputs arise from the deepest layer of the subiculum (McKenna and Vertes, 2004; Mathiasen et al., 2019), resulting in a partial overlap with subiculum cells that project to the anterior thalamic nuclei. Like the anterior thalamic nuclei, nucleus reuniens also receives sparse inputs from CA1 (Cenquizca and Swanson, 2006). However, in marked contrast, nucleus reuniens has dense, direct projections that terminate across CA1, alongside inputs to the ventral subiculum, entorhinal and perirhinal cortices (Herkenham, 1978; Wouterlood et al., 1990; Varela et al., 2014; see Fig. 4). A feature of the hippocampal inputs is that they are much denser in the ventral, rather than dorsal, CA1 (Hoover and Vertes, 2012; Valera et al., 2014).

**Fig. 3:**
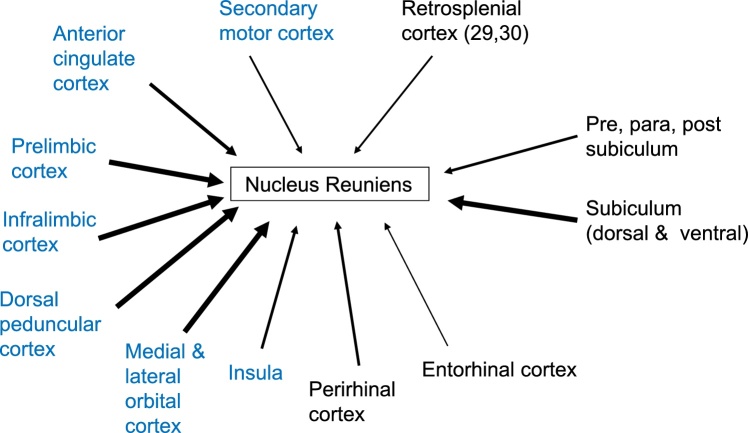
Cortical (and allocortical) inputs to nucleus reuniens. The sites in blue have few, if any, direct projections to the hippocampal formation. The thickness of the line represents the scale of the input.

**Fig. 4:**
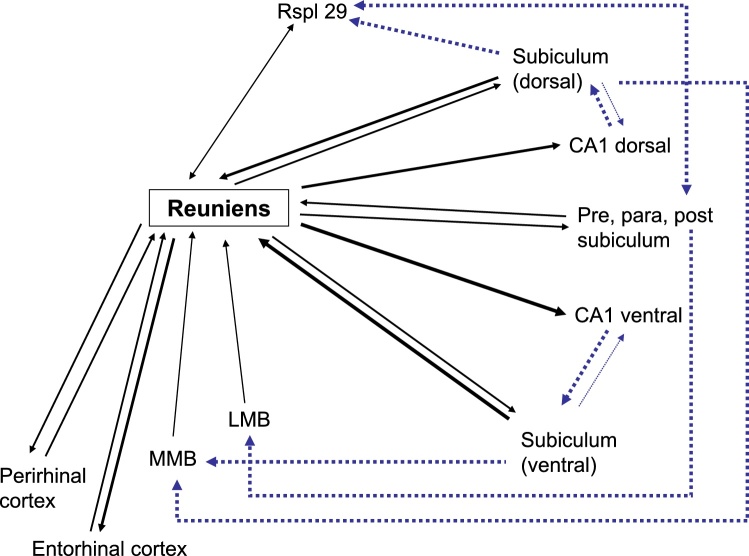
Schematic diagram of the main connections of the hippocampal formation and parahippocampal region with nucleus reuniens. The thickness of the line indicates the scale of the input, while the lightest connections are not shown. The indirect connections (dotted blue lines) involving retrosplenial cortex and the mammillary bodies are shown, reflecting their significance for anterior thalamic function. Note, not all connections are shown, just those most pertaining to the ATN and nucleus reuniens. Abbreviations: LMB, lateral mammillary nucleus; MMB, medial mammillary nucleus; para, parasubiculum; post, postsubiculum; pre, presubiculum; Rspl, retrosplenial cortex, area 29.

Nucleus reuniens, like the anterior thalamic nuclei, also receives subcortical inputs from the laterodorsal tegmental nucleus, raphe nucleus, reticular thalamic nucleus, and the peripeduncular tegmental nucleus (Herkenham, 1978; McKenna and Vertes, 2004). It also receives very light inputs from the mammillary bodies (McKenna and Vertes, 2004; Mathiasen et al., 2020). However, unlike the anterior thalamic nuclei, nucleus reuniens also receives inputs from numerous other subcortical sites, including the lateral septum, claustrum, substantia innominata, medial nucleus of the amygdala, paraventricular and lateral geniculate thalamic nuclei, zona incerta, multiple hypothalamic nuclei, the superior colliculus, pretectal nuclei, and the parabrachial nucleus (McKenna and Vertes, 2004). Consequently, compared to other thalamic nuclei, nucleus reuniens receives an unusually large array of ascending inputs (McKenna and Vertes, 2004). There is, however evidence that the mouse nucleus reuniens receives a more restricted set of subcortical inputs, with an apparent lack of afferents from the bed nucleus of stria terminalis, the amygdala, habenula, and some hypothalamic nuclei (Scheel et al., 2020).

Meanwhile, efferents from nucleus reuniens include a relatively dense projection to the claustrum (Vertes et al., 2006). Nucleus reuniens also has light, but diffuse, projections to a variety of subcortical sites, including the olfactory tubercle, preoptic area, lateral hypothalamic regions, the amygdala, medial and lateral septum, reticular thalamic nucleus, (Herkenham, 1978; Vertes et al., 2006). In addition, light projections to nucleus accumbens and the supramammillary nucleus have been described (Vertes et al., 2006). Other inputs to the pretectum and superior colliculus, ventral tegmental area and central grey, initially described by Herkenham (1978), may reflect projections from adjacent sites (Vertes et al., 2006).

### 2.3 Anterior thalamic nuclei versus nucleus reuniens connectivity

**Fig. 5:**
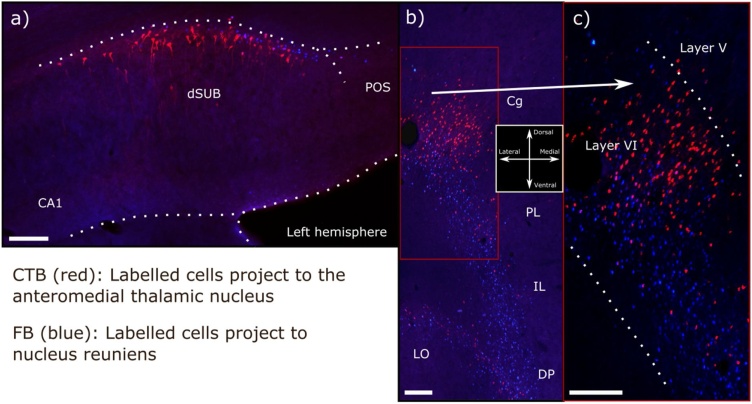
Photomicrographs of retrograde cell label resulting from tracers injected into nucleus reuniens and the anterior thalamic nucleus in the same brain. The tracer cholera-toxin b (CTB, red labelled cells) was infused into the anteromedial thalamic nucleus while fast blue (FB, blue labelled cells) was placed in nucleus reuniens. **a**) Distribution of retrograde labelled cells in the left dorsal subiculum. Despite the two cell populations being found in the deep cellular portion of the subiculum, the projections to nucleus reuniens tend to originate from deeper levels than the projections to the anteromedial thalamic nucleus. **b**) Distribution of retrograde labelled cells in the medial prefrontal cortex. The projection to the anterior thalamic nucleus originates predominantly from dorsal portions (anterior cingulate and dorsal prelimbic cortex) while cells that target nucleus reuniens are distributed along the entire dorsal-ventral axis. Additionally, these latter cells tend to be located at deeper portions of layer VI than cells projecting to the anteromedial thalamic nucleus. **c**) Higher resolution photomicrograph of the area indicated by a box in b). Abbreviations: CA, cornu ammonis, Cg, anterior cingulate cortex; CTB, cholera-toxin b; dSUB, dorsal subiculum; DP, dorsal peduncular cortex; FB, fast blue; IL, infralimbic cortex; LO, lateral orbitofrontal cortex; PL, prelimbic cortex; POS, postsubiculum. Scale bars =200 μm.

Despite numerous overlaps, there are key differences between the anterior thalamic nuclei and nucleus reuniens concerning their hippocampal interactions (Figs. 2, 4 and 5). Perhaps, the most obvious difference are the dense, direct projections from nucleus reuniens to the CA1, which are most dense in the ventral hippocampus (Herkenham, 1978; Varela et al., 2014). Consequently, nucleus reuniens provides a monosynaptic link between medial prefrontal cortex and CA1 (Vertes et al., 2007; Prasad and Chudasama, 2013). In addition, nucleus reuniens provides strong projections to both medial and lateral entorhinal cortex, as well as perirhinal cortex (Wouterlood et al., 1990). These links are all the more noteworthy as the prefrontal cortex has few, if any, direct projections to the hippocampal formation. At the same time, a subpopulation of nucleus reuniens neurons bifurcate and project to both frontal areas and CA1 (Hoover and Vertes, 2012; Varela et al., 2014), although separate nucleus reuniens cells innervate hippocampal and parahippocampal sites (Dolleman‐Van Der Weel and Witter, 1996).

The reverse connections (hippocampal formation to thalamus) also differ, but in a less obvious manner (Fig. 2, Fig. 4). While the subiculum projects to both thalamic sites, the inputs to nucleus reuniens principally arise from the very deepest cell population, adjacent to the alveus, and include polymorphic cells (Herkenham, 1978; McKenna and Vertes, 2004; Mathiasen et al., 2019). The subiculum neurons projecting to the anterior thalamic nuclei are also located deeply, frequently originating from pyramidal cells (Fig. 5). While the dorsal subiculum principally projects to the anterior thalamic nuclei, both the dorsal and ventral subiculum innervate nucleus reuniens (Meibach and Siegel, 1977b; Herkenham, 1978; Christiansen et al., 2016).

There are also clear differences in the relative densities of the various cortical inputs to nucleus reuniens and the anterior thalamic nuclei (Fig. 1, Fig. 2). While nucleus reuniens receives the majority of its cortical inputs from the prelimbic, rostral anterior cingulate, infralimbic, dorsal peduncular, lateral and medial orbital cortices, the anterior thalamic nuclei receive relatively more inputs from the more caudal anterior cingulate cortex and the retrosplenial cortex (areas 29 and 30) (Herkenham, 1978; Shibata, 1998; McKenna and Vertes, 2004; Shibata and Naito, 2005;Vann et al., 2009; Prasad and Chudasama, 2013; Wright et al., 2013). The inputs from these cortical sites to both nucleus reuniens and the anterior thalamic nuclei originate predominantly from layer VI, with much lighter inputs from layer V. While the majority of cortical cells that project to nucleus reuniens seem positioned very deep in layer VI (McKenna and Vertes, 2004; Herkenham, 1978), this pattern is not so evident for cells that projects to the anterior thalamic nuclei (Shibata and Naito, 2005, see also Fig. 5). Lastly, the perirhinal and insular cortices project more densely to nucleus reuniens than the anterior thalamic nuclei (Herkenham, 1978; McKenna and Vertes, 2004; Shibata and Naito, 2005; Wright et al., 2013).

As already noted, there are differences in the subcortical inputs to these two thalamic sites. Foremost are the very dense, unidirectional efferents from the mammillary bodies to the anterior thalamic nuclei (Shibata, 1992; Hopkins, 2005) that contrast with the much lighter mammillary body inputs to nucleus reuniens (Herkenham, 1978; McKenna and Vertes, 2004; Mathiasen et al., 2019). The significance of this input difference is signalled by how cutting the mammillothalamic tract (which innervates the anterior thalamic nuclei) is sufficient to impair many spatial learning tasks (Vann and Aggleton, 2003; Winter et al., 2011; Vann, 2013), including water-maze tasks sometimes spared after nucleus reuniens lesions (see Section 3.1). In contrast, as described in Section 2.2, nucleus reuniens receives inputs from a wide range of other subcortical sites that are not thought to project to the anterior thalamic nuclei (McKenna and Vertes, 2004).

The anterior thalamic nuclei have remarkably few, if any, descending projections, e.g., they do not seem to project back to the laterodorsal tegmental nucleus (Cornwall et al., 1990a; Satoh and Fibiger, 1986), the raphe nuclei, or periaqueductal grey matter (Peyron et al., 1997; Behzadi et al., 1990; Beitz, 1982). Perhaps their only subcortical projections are those within the thalamus, as part of their reciprocal connections with the reticular thalamic nucleus (Cornwall et al., 1990b; Gonzalo-Ruiz and Leiberman, 1995; Lozsadi, 1995). In contrast, nucleus reuniens projects to a variety of subcortical sites, albeit lightly, often appearing to reciprocate its afferent inputs (see Section 2.2). These projection sites include the septum, claustrum, preoptic area, amygdala, medial and lateral hypothalamic regions (Vertes et al., 2006).

### 2.4 Interim summary

Nucleus reuniens is a site of convergence from multiple subcortical areas that regulate its two-way role in linking frontal cortices with the hippocampus (McKenna and Vertes, 2004; Cassel et al., 2013; Griffin, 2015). In contrast, the topographic connections of the anterior thalamic nuclei not only help to establish the independence of its three major nuclei, but also enable the segregation of information in and out of the region. For example, the exceptionally dense inputs from the mammillary bodies to the anterior thalamic nuclei are topographically organised (Hayakawa and Zyo, 1989; Shibata, 1992), while very few afferent neurons innervate more than one anterior thalamic nucleus (Wright et al., 2013). This pattern appears consistent with the concept of a ‘first’ order thalamic nucleus (Sherman, 2007) but see Perry and Mitchell, 2019. This segregation is again seen by how the proximal subiculum innervates the anteromedial nucleus while the distal subiculum innervates the anteroventral nucleus (Meibach and Siegel, 1977b; Christiansen et al., 2016).

The contrasting distributions of subcortical connections (very limited for the anterior thalamic nuclei, more widespread for nucleus reuniens) reinforce the notion that anterior thalamic function is focussed on a restricted number of processes involving common aspects of information. Meanwhile, nucleus reuniens activity is influenced by a remarkable number of subcortical sites, alongside more limited descending projections, which together are consistent with a broader role in setting and guiding selection or control mechanisms (Dolleman et al., 2019.

The anterior thalamic afferents have a particular affinity with the dorsal half of the hippocampal formation (Fig. 2). Not only do the subiculum neurons projecting to the anterior thalamic nuclei principally originate in the dorsal hippocampus (Meibach and Siegel, 1977b; Christiansen et al., 2016), but the densest hippocampal inputs to retrosplenial cortex (especially area 29) also arise from the same dorsal subiculum regions (Naber and Witter, 1998; Kinnavane et al., 2018). In turn, area 29 has dense, reciprocal connections with the anteroventral and anterodorsal thalamic nuclei (Sripanidkulchai and Wyss, 1986; Shibata, 1998). While the anteroventral nucleus projects to the dorsal subiculum, the anteromedial nucleus projects to the ventral subiculum (Shibata, 1993; Van Groen and Wyss, 1995).

In contrast, nucleus reuniens has a greater affinity with the ventral half of the hippocampal formation (Fig. 4). The hippocampal inputs to nucleus reuniens originate mainly from the ventral subiculum (McKenna and Vertes, 2004), although there are topographic differences with dorsal reuniens receiving more dorsal hippocampal inputs, while ventral reuniens has more inputs from ventral hippocampal areas (Herkenham, 1978). The return projections predominantly arise from dorsal nucleus reuniens to (Dolleman‐Van Der Weel and Witter, 1996), terminating in both the dorsal and ventral CA1, but showing a strong preference for ventral CA1 (Herkenham, 1978; Prasad and Chudasama, 2013; Varela et al., 2014). Consequently, when using transynaptic tracers, it was observed that nucleus reuniens provides a monosynaptic link from more ventral frontal areas to more ventral parts of the hippocampal formation (Prasad and Chudasama, 2013). In contrast, more dorsal cortical areas (anterior cingulate and retrosplenial) project via the anterior thalamic nuclei to more dorsal hippocampal areas (Prasad and Chudasama, 2013).

These dorsal - ventral hippocampal gradients are of much interest given the consensus for functional changes along this hippocampal axis. While the dorsal (posterior) hippocampal formation is more allied to fine spatial and contextual distinctions, the ventral (anterior) hippocampal formation is more allied to lower spatial selectivity, potentially linked to greater object-based information (Ranganath and Ritchey, 2012; Poppenk et al., 2013; Strange et al., 2014). Other axial changes concern the greater interplay of the ventral hippocampal formation with hypothalamic and amygdala nuclei, suggestive of greater roles in anxiety and emotion (O’Mara, 2005; Aggleton, 2012; Strange et al., 2014). Consistent with this pattern, nucleus reuniens (unlike the anterior thalamic nuclei) has direct connections with multiple hypothalamic nuclei and the amygdala.

## 3 Respective lesion effects

### 3.1 Spatial learning

While lesions in both nucleus reuniens and the anterior thalamic nuclei can impair the learning and retention of spatial tasks that depend on the hippocampus (Aggleton and Nelson, 2015; Dolleman-van der Weel et al., 2019), their respective connections suggest that the outcomes will be qualitatively different. If just based on their hippocampal inputs and outputs, it might be supposed that lesions of nucleus reuniens would be the more disruptive, given its dense projections to CA1, which are not shared by the anterior thalamic nuclei. In fact, the opposite often appears to be the case. This outcome presumably reflects the different dorsal/ventral hippocampal alliances of these two nuclei, combined with the much greater mammillary body inputs to one site, set against the greater prefrontal interactions for the other. As a result, the anterior thalamic nuclei are more involved in spatial mapping and navigation, while nucleus reuniens helps to regulate and control that spatial information.

Comparing the consequences of anterior thalamic and nucleus reuniens lesions on spatial tasks is, unfortunately, not straightforward. One problem has been the prevailing use of different behavioural protocols when studying the two areas. A second issue concerns the challenge of making truly selective lesions in either thalamic site. Unsurprisingly, lesions that extend beyond the anterior thalamic nuclei can be more disruptive to spatial learning (e.g., Warburton et al., 1999). Meanwhile, for nucleus reuniens, surgeries often involve immediately adjacent thalamic areas, e.g., the rhomboid and submedial nuclei. Furthermore, many of the studies examining nucleus reuniens have used temporary inactivation methods, while this approach has rarely been used to analyse the anterior thalamic nuclei.

The impact of lesions in both the anterior thalamic nuclei and nucleus reuniens have been separately reviewed (e.g., Aggleton and Nelson, 2015; Dolleman-van der Weel et al., 2019), the task here is to contrast lesion effects on comparable spatial tasks, wherever possible. The circular Morris water-maze, designed to tax allocentric spatial memory, provides such an opportunity. While lesions in both thalamic target areas can disrupt spatial learning in the Morris water-maze, their effects are quite different. Anterior thalamic lesions severely impair initial location learning so that the rats take much longer to find the escape platform, while also failing to show a preference for that location when the platform is removed in probe trials (Sutherland and Rodriguez, 1989; Warburton and Aggleton, 1998; Warburton et al., 1999; Wolff et al., 2008; Moreau et al., 2013). In contrast, lesions or inactivation of nucleus reuniens can spare initial place learning, often only inducing performance deficits when task conditions are changed (Dolleman-van der Weel et al., 2009; Loureiro et al., 2012; Cholvin et al., 2013).

The severe deficits seen in the water-maze after anterior thalamic lesions could result from a failure to learn the correct location, a failure to navigate to that location, or both. In fact, both functions are disrupted. Severe location learning deficits are seen in tasks such as spontaneous object-in-place learning (Wilton et al., 2001; see also Nelson and Vann, 2014), contextual fear conditioning (Lopez et al., 2018), and object-place conditional learning (Sziklas and Petrides, 1999; Dumont et al., 2014a), all tasks that involve limited navigation. At the same time, disruption of the head-direction system following anterior thalamic lesions is thought to impair the monitoring of self-movement cues (Frohardt et al., 2006; Winter et al., 2011). Lastly, anterior thalamic lesions impair geometric learning in a modified water maze (Aggleton et al., 2009; Dumont et al., 2014b). In summary, anterior thalamic lesions cause a multiplicity of spatial deficits that result in poor allocentric learning, coupled with navigation problems. For these reasons it is assumed that all three anterior thalamic nuclei contribute to water-maze performance (van Groen et al., 2002).

In contrast, rats with lesions of nucleus reuniens can acquire the standard allocentric place learning problem in a Morris water-maze in a seemingly normal manner (Dolleman-van der Weel et al., 2009; Loureiro et al., 2012). On probe trials, when the escape platform was removed for the first time, rats with reuniens lesions headed correctly to the escape platform location but then searched more extensively across a wider area than control rats (Dolleman-van der Weel et al., 2009). Nevertheless, quadrant preference during the probe trial did not differ significantly from the ‘sham’ controls (Dolleman-van der Weel et al., 2009). Meanwhile, following drug-free acquisition, transient inactivation of reuniens/rhomboid prior to a probe trial, in which the escape platform was removed, reduced platform crossings and time spent in the correct quadrant (Cholvin et al., 2013). Nevertheless, preference for the correct quadrant again remained above chance (Cholvin et al., 2013). In other studies, reversible inactivation of nucleus reuniens affected acquisition but left probe performance intact (Davoodi et al., 2009). A further water maze finding is of location deficits only emerging after extended retention intervals of many days (Loureiro et al., 2012). Finally, when using a working memory protocol in the water-maze (new escape location every session), reversible nucleus reuniens lesions again spared acquisition, but impaired performance with more extended retention intervals (Davoodi et al., 2009). The conclusion is that effective location learning can occur without nucleus reuniens, but normal flexibility after changes in protocol and the ability to perform accurately after extended delays can be compromised.

Further lesion comparisons can be made when considering spatial working memory tasks such as radial-arm maze (RAM) foraging and T-maze alternation. The RAM test has repeatedly been shown to be highly sensitive to anterior thalamic lesions, with marked deficits that persist throughout acquisition (Aggleton et al., 1996; Byatt and Dalrymple-Alford, 1996; Mitchell et al., 2002; Moran and Dalrymple-Alford, 2003; Mitchell and Dalrymple-Alford, 2006). In two studies, the effects of NMDA lesions involving both nucleus reuniens and rhomboid were tested in an 8-arm RAM, with delays imposed after the first four choices (Hembrook and Mair, 2011; Prasad et al., 2017). In one study, where repeat testing heightened interference, consistent deficits were observed (Hembrook and Mair, 2011). In the other, the lesions caused transient perseverative deficits in acquisition, but performance recovered so that no deficits were observed after retention delays of 10 and 30 min. (Prasad et al., 2017). This sparing contrasts with the persistent deficits seen after anterior thalamic lesions.

Spatial alternation has been repeatedly used to examine both the anterior thalamic nuclei and nucleus reuniens, but comparisons are not straightforward. Studies of the anterior thalamic nuclei have largely relied on a discrete trial protocol in which the rat is picked up and moved back to the start of the T-maze between the sample and test trials (spatial nonmatching-to-sample). Meanwhile, the large majority of nucleus reuniens studies have employed a continuous T-maze alternation procedure (Dolleman-van der Weel et al., 2019). The advantage of the latter is that enables simultaneous electrophysiological recording. A disadvantage is that it may be prone to more response-based strategies, e.g., running a figure of eight path.

Unsurprisingly anterior thalamic lesions consistently impair discrete trial T-maze alternation, with marked deficits apparent from the very first training session and with the shortest test intervals (Aggleton et al., 1995a,b, 1996, Aggleton and Nelson, 2015; Frost et al., 2020). These deficits persist over subsequent training sessions (Aggleton et al., 1995a,b, 1996; Warburton and Aggleton, 1998) and extended periods of time post-surgery (Aggleton et al., 2009). While increasing the retention interval causes accuracy to fall even further (Aggleton et al., 1995a,b, 1996), because of baseline differences with controls these retention data are hard to interpret. Silencing of dorsal hippocampal (subiculum) projections to the anterior thalamic nuclei is sufficient to impair T-maze alternation when rats are not permitted to use intra-maze cues (Nelson et al., 2020).

There is lesion evidence that all three anterior thalamic nuclei contribute to spatial working memory, although the anteroventral nucleus may be the most critical (Aggleton et al., 1996; Byatt and Dalrymple-Alford, 1996). Evidence that these severe deficits are not merely due to a loss of head-direction information comes from the lack of effect of lateral mammillary body lesions on RAM and T-maze working memory tasks (Vann, 2005; Vann and Dillingham, 2019). Such lesions should disconnect head-direction signals from the anterior thalamic nuclei (Sharp and Koester, 2008). In contrast, mammillothalamic tract lesions, which disconnect the lateral and medial mammillary nuclei projections to all three anterior thalamic nuclei, impair both RAM and T-maze working memory, seeming to particularly disrupt allocentric spatial processing (Vann and Aggleton, 2003; Nelson and Vann, 2014; Perry et al., 2018). These findings again highlight the significance of the anteroventral and anteromedial nuclei as the lesion effects following mammillothalamic tract lesions are markedly greater than those of disconnecting the anterodorsal nucleus from the lateral mammillary nucleus (Vann and Aggleton, 2004; Vann, 2005; Aggleton et al., 2010).

Meanwhile, rats with nucleus reuniens lesions have also been tested on T-maze alternation, but typically using a maze in which the choice arms are directly connected back to the start point, which is at the bottom of the central stem (Layfield et al., 2015; Viena et al., 2018; Dolleman-van der Weel et al., 2019). In one such study, transient muscimol lesions of the reuniens/rhomboid nuclei were sufficient to consistently impair alternation performance with a retention delay of 30 s (Layfield et al., 2015). Using similar protocols, alternation deficits were again found for all retention delays tested (shortest 30 s) following muscimol infusion, while procaine impaired alternation after 120 s delays (Viena et al., 2018). The muscimol infusions also induced repeated wrong turns, suggesting a loss of behavioural flexibility (Viena et al., 2018). Meanwhile, optogenetic inhibition of nucleus reuniens during the sample phase was found to decrease choice accuracy, but not when applied during the delay or choice phases (Maisson et al., 2018), indicative of a role during encoding. Finally, a radial maze has been used to test choices between two arms, one recently visited (Hembrook et al., 2012). Inactivation of reuniens/rhomboid nuclei led to delay independent deficits on this two-choice spatial nonmatching task (Hembrook et al., 2012).

It is evident that both permanent and temporary lesions of nucleus reuniens can produce marked spatial alternation (nonmatching) deficits, indicative of working memory deficits (see also Hallock et al., 2013). The question of how these alternation deficits differ from those after anterior thalamic lesions is currently not possible to resolve as there are key protocol differences. Consequently, it is the findings from the water-maze and RAM that most clearly show how anterior thalamic damage can have more pervasive effects, while reuniens lesions can lead to inflexible responding or poor strategy choice. This difference can again be seen in automated (lever-pressing) nomatching-to-position tasks. Inactivation of reuniens/rhomboid leads to severe deficits at delays as short of 1 s, indicative of a breakdown in task strategy that is not delay dependent (Hembrook et al., 2012). Meanwhile, anterior thalamic lesions cause delay-dependent deficits on a very similar automated nonmatching task (Aggleton et al., 1991).

Given the severity of the spatial deficits, especially those after anterior thalamic damage, it is helpful to consider the selectivity of their effects. While anterior thalamic lesions consistently impair spatial tests involving allocentric information, they spare tests of egocentric spatial reference memory (Mitchell and Dalrymple-Alford, 2006; Wolff et al., 2008; Clark and Harvey, 2016). Furthermore, severe deficits in learning object – place conditional relationships occur after anterior thalamic nuclei lesions, yet normal learning rates are observed when the spatial demands are replaced (Sziklas and Petrides, 1999; Dumont et al., 2014a). Finally, both anterior thalamic nuclei and nucleus reuniens lesions impair the recognition of novel object-in-place combinations yet spare spontaneous object recognition (Warburton and Aggleton, 1999; Wilton et al., 2001; Barker and Warburton, 2018; see also Nelson and Vann, 2014). Unfortunately, spontaneous tests of recognition and associative recognition are very poor at discriminating between levels of deficit (Ameen-Ali et al., 2015), so it cannot be determined whether these object-in-place impairments are comparable.

### 3.2 Nonspatial learning

There is considerable evidence that pathology in the anterior thalamic nuclei or the disconnection of these nuclei is a key contributor to diencephalic amnesia (Aggleton and Brown, 1999; Harding et al., 2000; Carlesimo et al., 2011) highlighting how, in the human brain, these nuclei are vital for nonspatial information. While nucleus reuniens is not typically associated with diencephalic amnesia, its strong prefrontal connections are indicative of potential contributions to a wide range of nonspatial functions.

Temporal processing has attracted attention given its importance for episodic-like memory. Anterior thalamic lesions can impair a variety of temporal tasks. Examples include temporal alternation (Célérier et al., 2000) and the discrimination of temporal sequences of odours (Wolff et al., 2006). While rats with anterior thalamic lesions can make accurate spontaneous recency discriminations between single objects (Mitchell and Dalrymple, 2005) they are impaired when multiple objects are presented (Dumont and Aggleton, 2013). Meanwhile, nucleus reuniens lesions can impair recency discriminations between single object-pairs (Barker and Warburton, 2015), indicative of a more severe deficit. In addition, silencing medial prefrontal inputs to nucleus reuniens can also disrupt odour sequence learning, an impairment interpreted a failure of working memory strategies, rather than of temporal context information (Jayachandran et al., 2019).

The respective roles of these two thalamic areas in aspects of attention have also been considered. While anterior thalamic lesions spare a visual vigilance task (Chudasama and Muir, 2001) they do affect the ability of rats to learn to attend to the rewarded stimulus dimension across a series of nonspatial discriminations (i.e., they fail to acquire an ‘intradimensional set’), yet the same animals show *superior* switching to a novel stimulus dimension (an ‘extradimensional shift’) (Wright et al., 2015). Remarkably, rats with anterior thalamic lesions are quicker at learning a discrimination that relies on a hitherto inconsistently rewarded stimulus feature (extradimensional shift), than the previous series of discriminations that involve distinguishing a common feature, e.g., textures (‘intradimensional set’). The result is a negative switch-cost (Wright et al., 2015). (Normal rats show a positive switch-cost.) The implication is that the intact anterior thalamic nuclei assist in engaging attention and learning associated with stimulus dimensions that have been reliably rewarded in the past (as lesions cause increased attention to poor predictors of reward). This conclusion is supported by recent DREADDS analyses of anterior cingulate – anterior thalamic interconnections, showing that their disruption again leads to a negative switch cost (Bubb et al., 2020).

Meanwhile, a study of combined reuniens/rhomboid lesions also used an attentional set protocol (Linley et al., 2016), although it differed markedly in the sequence of discrimination types. Here, the lesions impaired an initial reversal and the following intradimensional discrimination. The same rats showed a positive shift-cost (Linley et al., 2016), as expected in normal animals, but the opposite to that seen after anterior thalamic lesions. Consequently, the performance profile of the reuniens/rhomboid lesioned rats contrasts with the effects of anterior thalamic lesions, instead it partly resembles that seen after orbital frontal lesions in rats (McAlonan and Brown, 2003). It is also the case that lesions of nucleus reuniens can improve attention to the relevant stimulus in an automated nose-poke delayed spatial nonmatching-to-sample task (Prasad et al., 2017).

Taken together, it appears that the anterior thalamic nuclei can aid attention or learning about classes of stimuli previously linked with reward, maintaining previously established strategies. Meanwhile there is evidence that nucleus reuniens, in concert with prefrontal cortices, can help promote flexible learning and behaviour (Dolleman-van der Weel et al., 2019). One predicted consequence is that for some competing aspects of attention and performance (Pearce and Mackintosh, 2010) there will be double dissociations between the impact of anterior thalamic and nucleus reuniens damage.

In addition to having potentially different roles in attentional mechanisms, there is growing reason to believe that nucleus reuniens makes further contributions of the processing of anxiety and fear. As already noted, nucleus reuniens has greater direct and indirect interconnections with hypothalamic nuclei and the amygdala than the anterior thalamic nuclei (Sections 2.3). Davoodi et al. (2011) initially showed that inactivation of nucleus reuniens need not affect acquisition of a passive avoidance task but can disrupt consolidation and retrieval. Subsequent studies have shown that nucleus reuniens and its medial prefrontal afferents are required for fear conditioning and its normal extinction in rats (Ramanathan and Maren, 2019; Ramanathan et al., 2018), while other evidence points to a role in regulating fear memory intensity and maintenance (Troyner et al., 2018). It appears that nucleus reuniens may have a wide impact on fear-related tasks.

Given its importance for location learning, it is not surprising that anterior thalamic nuclei lesions disrupt contextual fear conditioning (Dupire et al., 2013; Marchand et al., 2014; de Lima et al., 2017), with some evidence for a greater role in initial acquisition (de Lima et al., 2017). There is also, however, evidence of a wider contribution as anterior thalamic lesions can impair auditory (i.e., nonspatial) fear conditioning in mice (Célérier et al., 2000) while anterior thalamic lesions in rabbits can impair the extinction and re-acquisition of auditory fear conditioning (Gabriel et al., 1983). Anterior thalamic lesions may also reduce anxiety related behaviour in open mazes (Dupire et al., 2013). Consequently, both thalamic sites can make contributions to fear learning, but the lack of matched studies limits comparisons.

### 3.3 Interim summary

There are both qualitative and quantitative differences between the impact of lesions in the two thalamic sites. Anterior thalamic lesions are more disruptive to spatial location learning, a difference perhaps seen most clearly in the apparent inability of rats with anterior thalamic lesions to locate the escape platform in a water-maze. These impairments contrast with ability of rats with nucleus reuniens lesions to escape successfully during initial acquisition and retain a preference for the correct pool quadrant. The greater impact of anterior thalamic damage partly reflects the involvement of these nuclei in multiple, core aspects of spatial learning and navigation (Aggleton et al., 2010). Instead, spatial deficits after nucleus reuniens lesions more typically emerge after extended retention delays or when levels of interference are heightened. The implication is that the anterior thalamic nuclei are integral to spatial learning itself, while nucleus reuniens is required for performance under changed or more taxing conditions, e.g., high interference (Hembrook et al., 2012; Mei et al., 2018). This effect of increased interference might be linked to the presence of nucleus reuniens cells that show activity predictive of a subsequent turn in a T-maze (Ito et al., 2015; see Section 4), i.e., activity that might combat interference.

At the same time, evidence for contrasting effects on tests of extradimensional shifts (namely a facilitation after anterior thalamic lesions) points to different roles in acquired aspects of attention. While the intact anterior thalamic nuclei may be part of a system promoting attention to those classes of stimuli that have been rewarded in the past, nucleus reuniens may help promote flexibility in attending to other stimulus categories, i.e., a more executive function. It is intriguing to suppose how disruption of the former (anterior thalamic) processes might exacerbate spatial learning deficits in stable environments. Meanwhile, for nucleus reuniens, their role in attention might not affect place location learning in a stable environment, but their executive contribution could assist with novel challenges, such as water-maze probe trials and for distinguishing between test sessions when being given working memory tests in the water-maze or radial maze.

## 4 Electrophysiological findings

Recent research has considerably extended our understanding of the range of spatial stimuli that can promote activity in the anterior thalamic nuclei and nucleus reuniens (O’Mara and Aggleton, 2019; Table 1). The presence of numerous head-direction cells within the anterodorsal nucleus has long been appreciated (Taube, 1995, 2007) but, more recently, head-direction cells have also been found in the anteroventral (Tsanov et al., 2011), anteromedial, and parataenial (Jankowski et al., 2015) nuclei. Additionally, head direction cells have been described in nucleus reuniens (Jankowski et al., 2014).

**Table 1:** Summary properties of the rodent anterior thalamic nuclei, nucleus reuniens, and prelimbic cortex. The behaviour section, which includes the outcome of lesions involving prelimbic cortex lesions in rats, relates to both permanent lesions and transient inactivations. Symbols: √ present (for electrophysiology) √√ reflects greater frequency of cells; - no apparent effect (in the case of lesions); X impaired following lesions; XX severely impaired following lesions, ↑ enhanced performance; → direction of projection. In addition, -/X mixed results (no apparent effect or impaired). A blank space indicates that data are lacking. Abbreviations: AD, anterodorsal nucleus; AM, anteromedial nucleus; ant, anterior; ATN, anterior thalamic nuclei; AV, anterodorsal nucleus; C, cortex; EDS, extradimensional shift; IDS, intradimensional shift; Re, nucleus reuniens; recog, spontaneous recognition test; thal, thalamic; 5CSRTT, 5-choice serial reaction time task;. Sources of anterior thalamic nuclei and nucleus reuniens data from main text. References for prelimbic data from: Aggleton et al., 1995b; Birrell and Brown, 2000; Brito et al., 1982; De Bruin et al., 1994, 2001; Ennaceur et al., 1997; Hok et al., 2005; Joel et al., 1997; Jones and Wilson, 2005; Kolb et al., 1994; Muir et al., 1996; Passetti et al., 2000; Porter and Mair, 1997; Warburton and Brown, 2010.

Anatomy (main connections)	Anterior Thalamic Nuclei	Nucleus Reuniens	Prelimbic Cortex (PL)
Cortical	Retrosplenial, anterior cingulate (both reciprocal with ATN)	Prelimbic, infralimbic/dorsal peduncular, orbital, rostral anterior cingulate (all reciprocal with Re)	Insula, ant cingulate, infralimbic, orbital (all reciprocal with PL)
Allocortical	Dorsal/intermediate subiculum → AM, AV	Subiculum → reuniens	Subiculum → PL
	AV → Dorsal subiculum	Reuniens → CA1 (esp. ventral), ventral subiculum	CA1 → PL
	AM → Ventral subiculum		
	Postsubiculum ↔ AD		
Subcortical	Mammillary body inputs		
	Reticular thal n. ↔ ATN	Widespread reciprocal subcortical connections	PL↔ Reuniens, MD
	Very few other inputs		
			PL→ ATN
			Amygdala ↔ PL
			Claustrum ↔ PL
**Electrophysiology:**			
Head-direction cells	AD√√ AM√ AV√	√	Not present
Place cells	AM√	√ (larger place fields than AM)	√ (motivation/ goal related)
Perimeter/Border cells	AM√	√	
Theta present	AV√	√ (some theta skipping)	√ (CA1 coupling)
**Behaviour:** (lesions)			
Spatial			
Water maze - acquis	XX	-/X	-/X
Water maze - probe	XX	-X	-/X
T-maze alternation*	XX	XX	-/X
Radial-arm maze	XX	-/X	-/X
Object-in-place	XX	XX	XX
Object location		-	-

Nonspatial			
Object recognition	-	-	-
Recency recognition	-/X	X**	X
5CSRTT	-	X	X
Set-shifting IDS	X	X	-
Set-shifting EDS	↑	-	X
Reversal learning	-/X	X	-

* Comparisons not available for the same spatial alternation tasks.

In addition, place cells have been recorded in nucleus reuniens, the anteromedial nucleus, and the parataenial nucleus (Jankowski et al., 2015). The place cells in nucleus reuniens appear less precise than those in other thalamic nuclei, having larger place-fields, and thus a lower spatial information content. Finally, cells responsive to borders or boundaries are found in nucleus reuniens, as well as in the anteromedial and parataenial nuclei (Matulewicz et al., 2019). This discovery may relate to how anterior thalamic lesions impair the ability to use the geometric alignment of walls to determine a location (Aggleton et al., 2009; Dumont et al., 2014b), an ability not yet tested after nucleus reuniens lesions.

As already noted, place cells have not been reported in either the anterodorsal or anteroventral nuclei: but are present in reuniens, the anteromedial and parataenial nuclei (Jankowski et al., 2015). Thus, there are place cells present in the anterior thalamus, seemingly restricted to nuclei adjacent to nucleus reuniens; it is not known if this place information is shared between these nuclei, via, for example, local recurrent collaterals at the margins of these nuclei. At the same time, the possibility that spatial signals emerge at a much earlier stage of neural processing has, heretofore, received less attention than it might have done, in part because there have been relatively few electrophysiological explorations of these thalamic structures to date, despite strong evidence that subcortical structures can and do support spatial processing (see Jankowski et al., 2013; O’Mara and Aggleton, 2019).

Other relevant information comes from recordings of theta in the anterior thalamic nuclei (Vertes et al., 2001), which is most consistently found in the anteroventral nucleus. It has been reported (Tsanov et al., 2011) that about 40 % of head-direction cells in the rat anteroventral nucleus exhibit rhythmic spiking in the theta range (head direction-by-theta units). These units showed the greatest degree of theta rhythmicity when the animal was either heading or moving in the preferred direction of the cell. Moreover, approximately one-third of anteroventral thalamic units showing burst firing were also modulated by head direction (Tsanov et al., 2011). The crossover of both theta and head-directional firing suggests that the anteroventral nucleus processes information linking heading and movement. Meanwhile, some cells in nucleus reuniens fire in a synchronous or anti-synchronous relationship with theta cycles (Jankowski et al., 2014), so-called ‘theta-skipping’ cells. Such cells were first reported in the medial entorhinal cortex (mEC) (Brandon et al., 2013), where units can fire in a fixed synchronous or anti-synchronous relationship with alternate theta cycles. The theta-skipping cells in nucleus reuniens are not modulated by head-direction, and so might provide a pace-maker like function for synchronising some early components of the head-direction system (Jankowski et al., 2014).

A further contrast to be noted is that theta is present in nucleus reuniens, the anterodorsal and anteroventral nuclei, and modulates head directional firing. However, Jankowski et al. (2015), did not observe theta in the anteromedial or parataenial nuclei. It is not clear what the functional significance of the presence or absence of theta across these differing nuclei means, but one possibility is that theta serves a clock-like function in nucleus reuniens (Jankowski et al., 2014), and may serve additional attentional-modulation-like functions in the anteroventral nucleus (Tsanov et al., 2011).

To assess the wider significance of these thalamic spatial signals, several studies have measured the impact of selective thalamic lesions on spatial firing cells in hippocampal and parahippocampal areas. It was first shown that anterior thalamic lesions involving the anterodorsal nucleus cause a loss of head-direction firing in the postsubiculum (Goodridge and Taube, 1997). Subsequent studies found evidence for an even more widespread loss of the parahippocampal head-direction signal after anterior thalamic lesions and inactivations (Winter et al., 2015). That same study also reported how anterior thalamic lesions reduce the spatial periodicity of grid cells, as well as reducing the total numbers of grid cells, in the entorhinal cortex and parasubiculum (Winter et al., 2015).

In contrast, lesions involving the anterodorsal nucleus (i.e., affecting the head-direction signal) largely spare CA1 place cells as they continue to show location specific firing, although there is some loss of spatial coherence and information content, leading to greater place-field instability between sessions (Calton et al., 2003; see also Frost et al., 2020). Meanwhile, dramatic changes are seen in the subiculum as lesions of the anterior thalamic nuclei, both permanent and temporary, result in an apparent loss of all types of spatially responsive cell in the subiculum (Frost et al., 2020). This silencing includes place cells, despite the preservation of place cells in the adjacent CA1 field (Frost et al., 2020).

Parallel studies have shown that anterior thalamic lesions disrupt markers of activity and plasticity in many hippocampal subfields (Jenkins et al., 2002a,b; Dumont et al., 2012; Perry et al., 2018). These lesion effects, which typically reflect hypoactivity, are not just confined to those hippocampal areas that receive direct inputs from the anterior thalamic nuclei. Another indirect lesion effect concerns how mammillothalamic tract damage affects hippocampal – cortical oscillatory activity (Dillingham et al., 2019). Together, there is convergent evidence that anterior thalamic lesions and disconnections have appreciable, disruptive effects on a variety of related hippocampal and parahippocampal functions.

Information concerning the distal electrophysiological consequences of nucleus reuniens damage or inactivation is starting to amass (Dolleman-van der Weel et al., 2019). There is, for example, evidence that the medial prefrontal cortex, via nucleus reuniens, may help set future hippocampal path trajectories during goal directed behaviour (Ito et al., 2015). It has been found that in the continuous T-maze, units in nucleus reuniens (along with CA1 and medial prefrontal cortex) show ‘trajectory-dependent’ firing, i.e., they predict a subsequent left or right turn, while inactivation of nucleus reuniens reduces such firing in CA1 (Ito et al., 2015). At the same time, combined lesions of the rhomboid nucleus and nucleus reuniens (ReRh) spare the spatial characteristics of hippocampal CA1 place cells in familiar environments, although they disrupt their firing in unfamiliar contexts (Cholvin et al., 2018). Furthermore, recordings over a five-day period showed that ReRh lesions result in a marked and enduring decrease in place-field stability and altered firing variability (Cholvin et al., 2018). This pattern could be linked to the finding that lesions of nucleus reuniens often spare initial place learning in a water-maze but can lead to deficits after longer retention delays (Davoodi et al., 2009; Dolleman-van der Weel et al., 2009; Loureiro et al., 2012). From such findings it has been variously argued that nucleus reuniens (potentially with the rhomboid nucleus) contributes to the long term consolidation of memories that are initially hippocampal dependent (de Vasconcelos and Cassel, 2015) or that the head-direction signals of reuniens provide a stabilizing directional signal during the exploration of unfamiliar environments (O’Mara and Aggleton, 2019).

It is helpful to further contrast the functional differences of lesions to these thalamic nuclei. Frost et al. (2020) found that lesions of the anterior thalamic nuclei have combined behavioural and electrophysiological effects: spatial alternation performance drops to chance, *and* spatial signalling disappears in subiculum, but appears normal in CA1 (place cells appear unaffected by these lesions, as are control behavioural tests of recognition memory). Moreover, subicular spatial signalling is diverse (place, head direction, grid, speed, boundary vector: Anderson and O’Mara, 2004; Brotons-Mas, et al., 2017; Lever et al., 2009), i.e., the anterior thalamic lesions affected a wide range of spatial signals. By contrast, Cholvin et al. (2018) found that combined lesions of the rhomboid nucleus and nucleus reuniens appear to leave CA1 place cell spatial characteristics intact – but only when in familiar environments, i.e., abnormalities occurred when firing in unfamiliar environments. Rueniens/rhomboid lesions also induced a marked and lasting decrease in place field stability (Cholvin et al., 2018), although this experiment did not, however, include a behavioural assay. Nevertheless, it remains a logical possibility that the head directional signal from nucleus reuniens and the various spatial signals from the anterior thalamic nuclei provide sufficient spatial information for subicular spatial signalling *and* spatial alternation performance. Support for the former comes from a study transiently silencing anterior thalamic to dorsal hippocampal formation inputs (Nelson et al., 2020).

Thus, it may be that parallel circuits support consolidation of spatial processing. One (‘temporal lobe’) largely originates in entorhinal cortex (EC), projecting to DG, CA3, and CA1 (the trisynaptic circuit) and, thence, to cortical sites (e.g., EC and prelimbic), and is partly supported by nucleus reuniens, which provides a critical directional information to stabilise spatial hippocampal spatial information processing, prior to further cortical processing. Indeed, Hauer et al. (2019), suggest that nucleus reuniens has a critical role in co-ordinating slow-wave signals between hippocampus and prefrontal cortex [see also Dolleman-van der Weel et al. (2019) for a supporting discussion, and Roy et al. (2017) for data consistent with this possibility]. A separate critical pathway involves the anterior thalamic nuclei before reaching hippocampal (including subiculum) and parahippocampal areas, with further return connections (e.g., to mammillary bodies, anterior thalamic nuclei, retrosplenial cortex). Interactions between these systems may emerge at a cortical systems level – between prefrontal and retrosplenial cortices, as well as the hippocampal formation itself. The notion that these pathways emerge, in part, from different subcortical structures – the anterior thalamic nuclei and nucleus reuniens – and engage the hippocampus in different ways may help to explain how differing roles have been proposed for the hippocampus in memory (e.g. McClelland et al., 1995; Rolls, 1996; Nadel and Moscovitch, 1997).

## 5 Summary and conclusions

That the anterior thalamic nuclei and nucleus reuniens are interconnected with many of the same cortical sites points to complementary functions in overlapping domains. At the same time, the importance of the anterior thalamic nuclei for core aspects of hippocampal and parahippocampal spatial processing makes them the more critical for many spatial tasks (Table 1). Meanwhile, the connections of nucleus reuniens point to a more balanced role in spatial and nonspatial learning, which includes aspects of retrieval as well as initial learning.

These functional differences relate, in part, to the relative balance of dorsal (anterior thalamic) versus ventral (nucleus reuniens) hippocampal interconnections (Prasad and Chudasama, 2013; Fig. 2, Fig. 4), alongside the disproportionate contributions of the mammillary bodies to the anterior thalamic nuclei. This differential dorsal - ventral hippocampal gradient extends to their cortical connections, i.e., that the anterior thalamic nuclei are preferentially interconnected with the retrosplenial and anterior cingulate cortices, while nucleus reuniens is particularly interlinked with prelimbic, infralimbic, and orbital cortices (Fig. 1, Fig. 3). Consistent with these cortical differences is lesion evidence that nucleus reuniens contributes to executive and control behaviours (Cholvin et al., 2013; Prasad et al., 2013, 2017). For example, rats with nucleus reuniens lesions can show perseverative behaviour in spatial tasks (Viena et al., 2018), alongside abnormal impulse inhibition on the 5-choice reaction time task (Prasad et al., 2013), while anterior thalamic lesions seemingly spare performance of the same reaction time task (Chudasama and Muir, 2001).

These functional differences also relate to the relative balance of dorsal (anterior thalamic) versus ventral (nucleus reuniens) hippocampal interconnections (Prasad and Chudasama, 2013; Fig. 2, Fig. 4). The anteromedial and anteroventral thalamic nuclei receive dense inputs from the dorsal subiculum, while the postsubiculum and presubiculum innervate the anterodorsal nucleus. The pattern of segregated anterior thalamic connections (Wright et al., 2013) is consistent with processes able to operate at high levels of spatial resolution. Indeed, anterior thalamic cells have smaller place fields than those found in nucleus reuniens (O’Mara and Aggleton, 2019). Meanwhile, nucleus reuniens receives inputs from across the dorsal and ventral subiculum. It also projects throughout CA1, as well as the ventral subiculum. Anterior thalamic efferents also reach the subiculum (from the anteroventral and anteromedial nuclei), as well as the postsubiculum and presubiculum (from the anterodorsal nucleus).

It is reasonable to suppose that nucleus reuniens has its greatest hippocampal effects via activity on CA1, including its place cells (Cholvin et al., 2018). That analysis is consistent with evidence from disconnection studies highlighting the significance of medial frontal – CA1 interactions for spatial memory (Chao et al., 2017). Related evidence shows how inactivation of nucleus reuniens, as well as the medial prefrontal cortex and dorsal hippocampus, disrupts shifting from a response to a place strategy (Cholvin et al., 2013), i.e., an executive role. Meanwhile other research emphases the importance of nucleus reuniens – frontal interactions for spatial working memory (Griffin, 2015).

An issue running throughout this review concerns the different influences of the anterior thalamic nuclei and nucleus reuniens on the hippocampal formation. At its most simplistic this might be seen as a contrast between sensory (head-direction) and frontal control processes. The true picture is far more complex as both thalamic sites create indirect frontal – hippocampal pathways. Nevertheless, the role of a moderator between frontal areas and the hippocampal formation has been especially linked with nucleus reuniens. Aspects of this role are reflected in repeated evidence that nucleus reuniens is of particular importance for retrieval; i) after lengthy retention intervals, ii) when interference is high, and iii) when strategy flexibility is required, i.e., those same situations when prefrontal cortex is most vital for spatial memory tasks. Consequently, nucleus reuniens has a somewhat intermittent relationship with the hippocampus, largely set by its many cortical and subcortical inputs, which help determine when nucleus reuniens is needed to optimise consolidation and retrieval. In contrast, the anterior thalamic nuclei appear to operate continuously with the hippocampus to ensure accurate spatial encoding, initial consolidation, and aid retrieval.

At present, little is known about the roles of the many subcortical connections possessed by nucleus reuniens. In contrast, it has long been appreciated that in order to understand the anterior thalamic nuclei it is necessary to understand their mammillary bodies inputs (Vann and Aggleton, 2003; Nelson and Vann, 2014; Vann, 2009, 2013). One direction for future research will be to broaden the examination of the various subcortical inputs to both nucleus reuniens and to the anterior thalamic nuclei (Mitchell et al., 2002). Meanwhile, an overarching goal is to understand why there is apparent duplication between the hippocampal formation and the anterior/midline thalamic nuclei, given that both areas contain spatially responsive cells and both areas are critical for spatial learning and memory (O’Mara and Aggleton, 2019). It is increasingly evident that the different patterns of connectivity of these two thalamic structures provide one part of the answer, with the anterior thalamic nuclei vital for multiple aspects of spatial encoding and retention, while nucleus reuniens operates on that information to aid performance, especially under demanding or changing conditions.

## Declaration of Competing Interest

The authors report no declarations of interest.
